# Antioxidant and Wound Healing Property of Gelsolin in 3T3-L1 Cells

**DOI:** 10.1155/2020/4045365

**Published:** 2020-02-12

**Authors:** Bhavna Vaid, Bhupinder Singh Chopra, Sachin Raut, Amin Sagar, Maulik D. Badmalia, Ashish, Neeraj Khatri

**Affiliations:** ^1^IMTECH Centre for Animal Resources & Experimentation (iCARE), CSIR-Institute of Microbial Technology, Sector 39-A, Chandigarh 160036, India; ^2^Division of Protein Science & Engineering, CSIR-Institute of Microbial Technology, Sector 39-A, Chandigarh 160036, India

## Abstract

Delineation of factors which affect wound healing would be of immense value to enable on-time or early healing and reduce comorbidities associated with infections or biochemical stress like diabetes. Plasma gelsolin has been identified earlier to significantly enable injury recovery compared to placebo. This study evaluates the role of rhuGSN for its antioxidant and wound healing properties in murine fibroblasts (3T3-L1 cell line). Total antioxidant capacity of rhuGSN increased in a concentration-dependent manner (0.75-200 *μ*g/mL). Cells pretreated with 0.375 and 0.75 *μ*g/mL rhuGSN for 24 h exhibited a significant increase in viability in a MTT assay. Preincubation of cells with rhuGSN for 24 h followed by oxidative stress induced by exposure to H_2_O_2_ for 3 h showed cytoprotective effect. rhuGSN at 12.5 and 25 *μ*g/mL concentration showed an enhanced cell migration after 20 h of injury in a scratch wound healing assay. The proinflammatory cytokine IL-6 levels were elevated in the culture supernatant. These results establish an effective role of rhuGSN against oxidative stress induced by H_2_O_2_ and in wound healing of 3T3-L1 fibroblast cells.

## 1. Introduction

Any disruption in the normal structure and function of the skin and underlying soft tissue results in wound formation [1]. Soon after an injury occurs to a tissue, the process of healing begins in different phases to repair the wound. The healing process includes hemostasis, inflammation, proliferation (granulation), and remodelling (maturation and contraction) [2, 3]. Hemostasis is marked by vasoconstriction, platelet aggregation, and collagen adhesion to the basement membrane of the injured endothelial cells for initiating angiogenesis [4], which is imperative for maintaining nutrients and oxygen levels at the site of injury [5–7]. Subsequently, as inflammatory response, neutrophils and monocytes are recruited to the site of injury stimulating the release of proteolytic enzymes, proinflammatory cytokines, and growth factors. The proliferation phase is characterized by reepithelialization, neovascularization, and connective tissue formation through proliferation and migration of fibroblasts [8]. In order to fill the wound gap and rebuild the physical structure, fibroblasts mature into myofibroblasts, synthesizing and depositing the extracellular matrix (ECM), mainly collagen. Myofibroblasts also facilitate wound contraction and maturation of the granulation tissue [9]. The remodelling phase includes the reduction of cellularity of the tissue due to apoptosis of myofibroblasts, endothelial cells, and inflammatory cells and ultimately results in the synthesis of ECM [9, 10]. It is followed by remodelling of granulation tissue from immature connective tissue to mature connective tissue through extracellular collagen formation [11]. However, wound healing can be prolonged in a number of complicating comorbidities, and new medical interventions for improving wound healing are needed.

Gelsolin (GSN), an 85.7 kDa calcium-dependent protein, is known for its role in actin assembly regulation. Gelsolin controls actin metabolism by severing, capping, and nucleating actin [12]. Commonly, it has two forms: cytoplasmic and plasma, and their expressions are differentially regulated [13]. The plasma form has a signal sequence, which gets processed and allows the protein to be available extracellularly in mammals. It is the dominant actin depolymerizing factor (ADF) in plasma and directly/indirectly regulates rheodynamics of plasma. Plasma gelsolin (pGSN) thus plays a critical role in severing and clearing actin filaments released into the bloodstream after any tissue injury [14]. In addition, gelsolin levels have been stated to decline in a plethora of diseases, and supplementing exogenous recombinant human gelsolin (rhuGSN) alleviated distress symptoms in many disease conditions including sepsis, inflammation, diabetes [15, 16], thrombosis, and pulmonary thromboembolism [17]. Interestingly, cytoplasmic gelsolin (cGSN) can bind to globular actin to nucleate its polymerization and surprisingly can also bind to filamentous actin to break and cap it back to monomeric actin form [12, 18, 19].

The term “gelsolin” encompasses wild-type gelsolin, isoforms, analogs, variant, fragments, or functional derivatives of gelsolin as well as synthetic and recombinant gelsolin [18]. pGSN composed of about 800 amino acids is highly identical in all elements including the structure and function between mammals. In standard purification condition, the recombinant gelsolin from *E. coli* is analogous to natural human plasma gelsolin; the only difference is a disulfide bond that is present in the human plasma gelsolin. Also, the recombinant protein retains the same structural and functional characteristics if its purification is followed by oxidation [20]. This allowed other researchers and our team to use recombinant human gelsolin (rhuGSN) for various *in vivo* experiments [15–17, 21, 22]. Since in injury or cell death events, there is an increased influx of actin in circulating plasma and pGSN being the primary ADF, we wanted to test efficacy of this protein in the wound healing process and designed the current study. In this study, we have demonstrated the *in vitro* antioxidant activity along with wound healing property of rhuGSN in a scratch assay using a 3T3-L1 mouse embryo fibroblast cell line.

## 2. Materials and Methods

### 2.1. Chemicals, Drugs, and Reagents

Methods of expression, purification, and systematic characterization of rhuGSN were followed as described by us earlier [22–24]. Briefly, His-tag at N-terminal bearing gelsolin was expressed in *E. coli* in inducible format. Cells were lysed, and the protein was extracted from cytoplasm using a Ni-NTA-based affinity column followed by gel filtration. The purity and identity of protein were ascertained by expected migration in SDS-PAGE (followed by antigelsolin western blots) and MALDI-TOF, respectively. Furthermore, gelsolin was routinely characterized by its ability to depolymerize/nucleate F-actin and small angle X-ray scattering (SAXS) experiments in our group. Overall, purity, identity, and precise concentration of pGSN were done to required diligence before commencing experiments. Importantly, the protein was eluted through a Polymyxin B column to remove base levels of LPS to below the detection level before using samples for cell line experiments [22]. A total antioxidant capacity estimation kit (BioVision Inc., USA), CBA-Flex kit (BD Biosciences), DMEM (Genetix Biotech), Fetal Bovine Serum (FBS) (GIBCO), 3-(4,5-dimethylthiazol-2-yl)-2,5-diphenyltetrazolium bromide (Sigma), sodium dodecyl sulfate (Sigma), dimethylformamide (Sigma), and H_2_O_2_ (Merck) were used in the study.

### 2.2. Cell Line

The 3T3-L1 mouse embryo fibroblast cell line used for the *in vitro* scratch assay was procured from the National Centre for Cell Science (NCCS), Pune, India.

### 2.3. Antioxidant and Wound Healing Potential of rhuGSN *In Vitro*

#### 2.3.1. Total Antioxidant Capacity (TAC) of rhuGSN

Total antioxidant capacity of rhuGSN was determined using a colorimetric assay kit (BioVision Inc., USA) as per the manufacturer's instructions. Different concentrations of rhuGSN (0.75, 1.5, 3, 6.25, 12.5, 25, 50, 100, and 200 *μ*g/mL) were used, and a standard curve of known concentrations of Trolox was drawn against equivalent concentration, and finally, the total antioxidant capacity of rhuGSN was calculated.

#### 2.3.2. Cell Viability Assay

3T3-L1 cells (5 × 10^3^ cells/well) were cultured into a 96-well plate in DMEM culture medium with 10% FBS. Subsequently, different concentrations of rhuGSN (0.375, 0.75, 1.5, 3, 6.25, 12.5, 25, 50, 100, and 200 *μ*g/mL) were added to all the wells except blank and control wells that contained medium alone and cells in the medium, respectively. The culture plate was incubated at 37°C in a humidified CO_2_ incubator for 24 h. Later, cell viability was assessed as suggested by Mosmann [25] with few modifications. Twenty *μ*L of 3-(4,5-dimethylthiazol-2-yl)-2,5-diphenyltetrazolium bromide (MTT) solution (5 mg/mL in PBS) was added to each well, and the plate was incubated at 37°C in a humidified CO_2_ incubator for 4 h. Subsequently, 80 *μ*L of extraction buffer (20% sodium dodecyl sulfate in 50% dimethylformamide) was added to each well and incubated at 37°C in a humidified CO_2_ incubator for 4 h. The absorbance of formazan formed was determined at 570 nm using a BioTek ELISA plate reader. The percent cell viability was calculated and compared with respect to the control:

(1) $$\begin{array}{c} \%\text{Cell Viability}=\frac{\left(\text{Abs sample}-\text{Abs blank}\right)}{\left(\text{Abs control}-\text{Abs blank}\right)}\times 100. \end{array}$$

#### 2.3.3. Effect of rhuGSN on Hydrogen Peroxide- (H_2_O_2_-) Induced Oxidative Stress in 3T3-L1 Cells

The ability of rhuGSN in alleviating oxidative stress induced by hydrogen peroxide was studied in 3T3-L1 cells as described earlier [26–28]. Firstly, the inhibitory concentration of H_2_O_2_ was calculated using a cell viability assay as described above for various concentrations (0.01, 0.1, 1, 10, 100, and 1000 mM) of H_2_O_2_. In our experiment, a dose of 1 mM H_2_O_2_ could decrease the cell viability of 80% after 24 h of exposure and therefore was selected for further investigations. Various concentrations of rhuGSN (12.5, 25, 50, 100, and 200 *μ*g/mL) were used to treat cells in different regimens as follows:

1. Cells were exposed concomitantly to rhuGSN and 1.0 mM H_2_O_2_ for 24 h
2. Cells were exposed to 1.0 mM H_2_O_2_ for 3 h followed by treatment of cells with rhuGSN for 24 h
3. Cells were pretreated with rhuGSN for 24 h followed by 1.0 mM of H_2_O_2_ exposure for 3 h. Cell viability was checked using the MTT assay as described under procedure for cell viability

#### 2.3.4. Scratch Assay to Evaluate the Wound Healing Ability of rhuGSN

The stimulatory effect of rhuGSN on the migration of 3T3-L1 cells was determined as described by Pitz et al. [29]. 3T3-L1 cells (5 × 10^3^ cells/well) were cultured into a 24-well plate in DMEM culture medium with 10% FBS. The culture plate was incubated overnight at 37°C in a humidified CO_2_ incubator. After incubation, DMEM was completely removed and the adherent cell layer was scratched with a sterile 200 *μ*L pipette tip. Cellular debris was removed by washing off with phosphate buffer saline (PBS). The cells were treated with DMEM medium having different concentrations of rhuGSN (12.5, 25, 50, and 100 *μ*g/mL). Controls received only fresh DMEM, whereas DMEM supplemented with 10% FBS was taken as the positive control. Images of the scratch area (wound area) at 0 h were taken using a built-in camera in the microscope (40x magnification), and then, the plate was incubated at 37°C in a humidified CO_2_ incubator for 12 h. Alterations in the injured area after different time points (12, 16, 20, and 24 h) were again captured. Data were evaluated using TScratch imaging software (CSE Lab., ETH, Zurich) to calculate the percent wound area [30].

#### 2.3.5. Cytokine Profile

Culture supernatant was collected from all the wells from the scratch assay at 12 h and pooled for estimation of different cytokines. Levels of cytokines such as IL-2, IL-4, IL 6, IL-10, IL-17a, TNF-*α*, and IFN*γ* were measured by a CBA-Flex kit using BD FACSCalibur according to the manufacturer's instructions (BD Biosciences).

### 2.4. Statistical Analysis

The results are expressed as the mean ± SD. All statistical analyses were done using one-way ANOVA followed by the Student unpaired *t*-test. A value of *p* < 0.05 was considered statistically significant.

## 3. Results

### 3.1. Total Antioxidant Capacity (TAC) of rhuGSN

**Figure 1:**
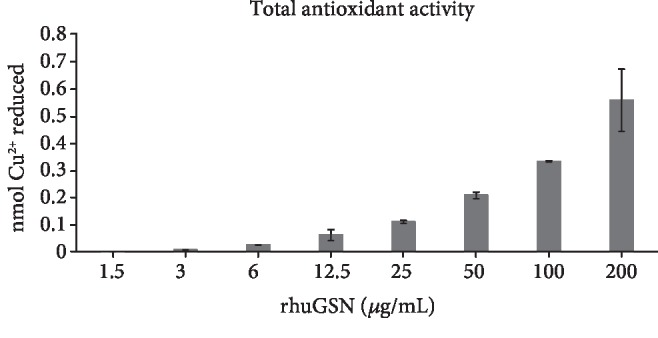
Total antioxidant capacity of rhuGSN in 3T3-L1 cells.

rhuGSN exhibited increased TAC in a concentration-dependent manner. The value of TAC for rhuGSN increased from 0.01 to 0.56 of nmol Cu^2+^ reduced for its lower concentration to higher concentration (Figure 1).

### 3.2. Cell Viability Assay

**Figure 2:**
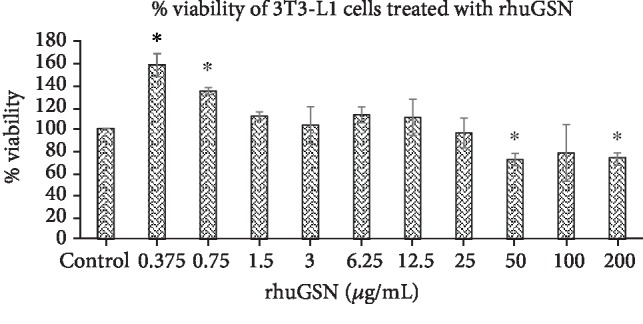
Percent viability of 3T3-L1 cells treated with rhuGSN. Data are expressed in mean ± SD. ∗ indicates *p* < 0.05 against the control after 24 h.

The results of the viability assay are summarized in Figure 2. rhuGSN exhibited significant viability (more than 70%) of cells at different concentrations (0.375-200 *μ*g/mL) tested for 24 h. Intriguingly, while at lower concentration a substantial and significant increase is observed in proliferation of cells, at higher concentration (50-200 *μ*g/mL), a marginal but significant decrease has been observed.

### 3.3. Hydrogen Peroxide-Induced Oxidative Stress in 3T3-L1 Fibroblast Cells

The antioxidant property of rhuGSN in hydrogen peroxide-induced oxidative stress was evaluated by the following:

- Cells were exposed concomitantly to rhuGSN and 1.0 mM H_2_O_2_ for 24 h
- Cells were exposed to 1.0 mM H_2_O_2_ for 3 h followed by treatment of cells with rhuGSN for 24 h
- Cells were pretreated with rhuGSN for 24 h followed by 1.0 mM of H_2_O_2_ exposure for 3 h

**Figure 3:**
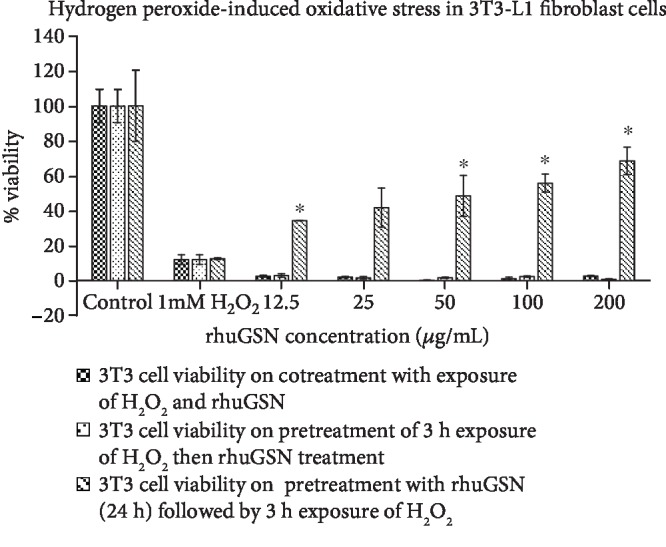
Percent viability of 3T3-L1 cells treated with rhuGSN. ∗ indicates *p* < 0.05 against 1.0 mM H_2_O_2_ control.

rhuGSN did not show any protection against oxidative stress when cells were exposed to H_2_O_2_ before or simultaneously with rhuGSN leading to the decrease in cell viability. On the other hand, pretreatment of cells with different concentrations (12.5-200 *μ*g/mL) of rhuGSN for 24 h followed by exposure to H_2_O_2_ for 3 h resulted in dose-dependent survival (35-70%) of cells (Figure 3).

### 3.4. Scratch Assay to Evaluate Wound Healing Capability of rhuGSN

**Figure 4:**
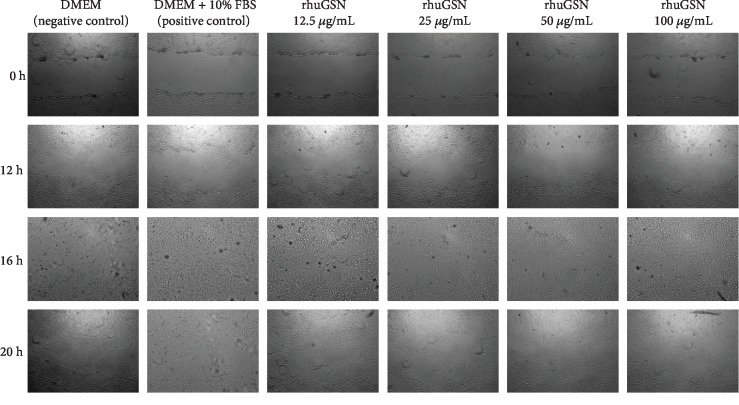
Microscopic images of the 3T3-L1 fibroblast cell wound area in the scratch assay after 0 h, 12 h, 16 h, and 20 h incubation.

**Figure 5:**
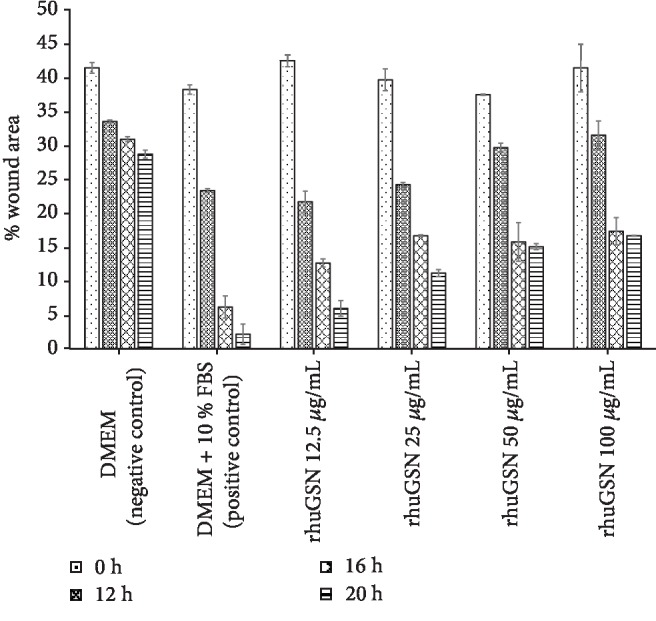
Wound area (%) in the scratch assay after 0 h, 12 h, 16 h, and 20 h post rhuGSN treatment. Data are expressed in mean ± SD.

3T3-L1 fibroblast cells (5 × 10^3^ cells/well) were treated with rhuGSN following induction of scratch to evaluate the wound healing capability of rhuGSN. Wound healing in cells was observed up to 24 h post scratching. Treatment with different concentrations of rhuGSN (12.5, 25, 50, and 100 *μ*g/mL) resulted in faster recovery as shown by the reduced wound area created by the scratch (Figures 4 and 5) after 20 h of injury as compared to 0 h. However, wound healing capability of rhuGSN at a concentration of 12.5 *μ*g/mL was even better as compared to that of other concentrations. Complete wound healing was observed in all the wells at 24 h of injury.

### 3.5. Cytokine Profile of the Culture Supernatant

**Figure 6:**
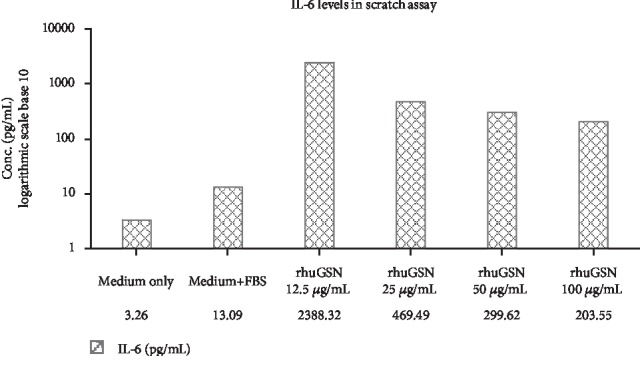
IL-6 cytokine levels in the culture supernatant from the scratch assay.

Levels of IL-6 increased in the culture supernatant treated with different concentrations of rhuGSN as compared to the control. The highest concentration of IL-6 was observed when the cells were treated with 12.5 *μ*g/mL rhuGSN (Figure 6). Levels of other cytokines such as IL-2, IL-4, IL-10, IL-17a, TNF-*α*, and IFN*γ* did not show any change as compared to the control group (data not shown).

## 4. Discussion

The purpose of this study was to explore the antioxidant and wound healing properties of gelsolin in the *in vitro* system using 3T3-L1 cells. There are two primary isoforms of gelsolin, intracellular or cytoplasmic gelsolin (cGSN) and an extracellular or plasma gelsolin (pGSN). Both forms are encoded by the same gene on chr 9. These isoforms are identical except for an additional 27 amino acid N-terminal signal peptide in pGSN [18]. GSN is expressed primarily in platelets, fibroblasts, and smooth and skeletal muscle cells. The cGSN plays an important role in maintaining cytoskeleton, whereas pGSN regulates the integrity of actin filaments by severing and capping [12]. rhuGSN is a recombinant form of pGSN having a similar structure and function as pGSN [18]. It is well established that the level of pGSN declines in a variety of ailments such as inflammation, diabetes, trauma, sepsis, rheumatoid arthritis, and multiple sclerosis [15, 16, 21, 31], and exogenous rhuGSN supplement/replacement therapy effectively rescues the affected animals from inflammation [15, 32], sepsis [9, 22], burn [33], diabetes [16], and thrombosis and pulmonary thromboembolism [17].

In the present study, we have demonstrated that rhuGSN (0.375-12.5 *μ*g/mL) increases cell viability and promotes fibroblast proliferation, leading to wound healing in 3T3-L1 cells. Our findings are consistent with previous reports demonstrating the proliferative effect of recombinant pGSN on human corneal epithelial cells and mesangial cells [34–36]. The rhuGSN also exhibited antioxidant property by protecting the cells from oxidative stress induced by H_2_O_2_ exposure. Oxidative stress plays a key role in the wound healing process as reactive oxygen species (ROS) generated as a defence mechanism may inhibit cell proliferation [37]. In fact, we found here that pretreatment with rhuGSN protected 3T3-L1 cells from oxidative stress induced by H_2_O_2_. Notably, plasma gelsolin is also known to play an important role in severing and clearing actin filaments released upon any tissue injury [14, 38, 39]. It is likely that in our *in vitro* wound healing model, rhuGSN cleared the actin filaments that were released upon oxidative stress and promoted wound healing.

Cell proliferation and migration are two essential characteristics of the wound healing process. The healing process is mimicked by the scratch assay *in vitro.* Disruption of the cell monolayer leads to loss of cell-cell contact, which after aggregation and release of growth factors/cytokines at the wound surface enhances cell migration and proliferation [40]. Gelsolin improves the cell viability in human monocyte cells (THP-1) when cocultured with polyethylene, titanium, and cobalt particles and LPS [41]. In our experiments, rhuGSN promoted proliferation and migration of fibroblasts and caused wound closure with the production of cytokine IL-6. IL-6 and IL-10 have been reported to protect the epithelial barrier and enhance the reepithelialization process in wound healing [42, 43]. IL-6 stimulation has also been reported to fasten the closure of wound in rabbits and human corneal cells [44, 45]. Earlier studies have reported the ability of gelsolin to trigger proinflammatory cytokine secretion (e.g., TNF- *α*, IL-6, and IL-1*β*) from human monocyte cells [41]. Considering these observations, we hypothesised that increased IL-6 concentration in the culture supernatant upon rhuGSN supplementation might have facilitated the early closure of wound in our *in vitro* fibroblast cell culture model. Interestingly, gelsolin also exhibits anti-inflammatory properties, and its ability to modulate the polymeric state of actin is pivotal for cell proliferation and final topology of cell-cell layering [46]. In an ocular surface regeneration model, recombinant plasmatic gelsolin therapy promoted wound healing by acting on the epithelial cells as well as in deeper corneal layers such as the stroma, where fibroblasts are differentiated into myofibroblasts [34]. The role of gelsolin as actin regulatory protein is well established [15, 47, 48]. Gelsolin also acts as a buffering agent in inflammation by binding to LPS, platelet-activating factor (PAF), and lysophosphatidic acid (LPA) thereby sequestering the bioactive mediators of inflammation and limiting the inflammatory and immune reactions [49]. In addition to scavenging and the counterbalancing role of pGSN in inflammation, it is also essential for the regulation of rheodynamics of the cellular microenvironment, promoting an especially suitable physicochemical condition for greater cell migration and wound healing [50]. During the wound healing process, fibroblast and macrophages release FliI (a gelsolin family protein), which can be upregulated upon wounding [51]. Accordingly, addition of rhuGSN to fibroblast culture promoted actin cytoskeletal remodelling, which supported cell proliferation and migration and promoted IL-6 secretion necessary for early wound healing.

Interestingly, the protective effects of rhuGSN observed for oxidative stress and injury plateaued at 25 *μ*g/mL and beyond. Furthermore, while the lower concentration of rhuGSN led to substantial increase of IL-6, a marginal decrease is observed at 25 *μ*g/mL and beyond. The plateauing of wound repair capability, reduction in oxidative stress, and slight decrease in proliferation observed at higher concentration of rhuGSN could be ascribed to a concomitant marginal decrease observed in IL-6, which has a reported role in cell proliferation as well as wound healing [34]. However, the *in vitro* assays cannot truly represent the complex wound healing process *in vivo*; therefore, further validation of this study in animal models would potentially help in establishing the role of rhuGSN in wound healing.

## 5. Conclusion

In this study, we have demonstrated the antioxidant and wound healing properties of rhuGSN in 3T3 cells. rhuGSN showed cytoprotection following oxidative stress induced by H_2_O_2_ exposure. In the wound healing scratch assay, rhuGSN boosted IL-6-mediated wound healing by promoting proliferation and migration of fibroblast cells.

## Data Availability

The data used to support the findings of this study are included within the article.
